# Machine-learning based feature selection for a non-invasive breathing change detection

**DOI:** 10.1186/s13040-021-00265-8

**Published:** 2021-07-18

**Authors:** Juliana Alves Pegoraro, Sophie Lavault, Nicolas Wattiez, Thomas Similowski, Jésus Gonzalez-Bermejo, Etienne Birmelé

**Affiliations:** 1 UMR CNRS 8145, Laboratoire MAP5, Université de Paris, 45 rue des Saints-Pères, Paris, 75006 France; 2 Sorbonne Université, INSERM, UMRS1158 Neurophysiologie Respiratoire Expérimentale et Clinique, Paris, F-75005 France; 3 SRETT, 11 Rue Heinrich, Boulogne-Billancourt, 92100 France; 4 AP-HP, Groupe Hospitalier Universitaire APHP-Sorbonne Université, site Pitié-Salpêtrière, Service de Pneumologie, Médecine Intensive et Réanimation (Département R3S), Paris, F-75013 France; 5 Institut de Recherche Mathématique Avancée, UMR 7501 Université de Strasbourg et CNRS, 7 rue René-Descartes, Strasbourg, 67000 France

**Keywords:** Respiratory pattern, Telemonitoring, Classification, Novelty detection, Chronic obstructive pulmonary disease (COPD)

## Abstract

**Background:**

Chronic Obstructive Pulmonary Disease (COPD) is one of the top 10 causes of death worldwide, representing a major public health problem. Researchers have been looking for new technologies and methods for patient monitoring with the intention of an early identification of acute exacerbation events. Many of these works have been focusing in breathing rate variation, while achieving unsatisfactory sensitivity and/or specificity. This study aims to identify breathing features that better describe respiratory pattern changes in a short-term adjustment of the load-capacity-drive balance, using exercising data.

**Results:**

Under any tested circumstances, breathing rate alone leads to poor capability of classifying rest and effort periods. The best performances were achieved when using Fourier coefficients or when combining breathing rate with the signal amplitude and/or ARIMA coefficients.

**Conclusions:**

Breathing rate alone is a quite poor feature in terms of prediction of breathing change and the addition of any of the other proposed features improves the classification power. Thus, the combination of features may be considered for enhancing exacerbation prediction methods based in the breathing signal.Trial Registration : ClinicalTrials NCT03753386. Registered 27 November 2018, https://clinicaltrials.gov/show/NCT03753386

## Background

### Motivation

Chronic Obstructive Pulmonary Disease (COPD) is one of the top 10 causes of death worldwide, representing a major public health problem [1]. It is characterized by permanent and progressive obstruction of the airways, which may result in an accelerated decline in respiratory function. Increasing breathing difficulty often leads to a reduction in daily activities and a deterioration in the quality of life.

Besides, patients with COPD may experience periods of acute deterioration of symptoms, called exacerbations. Exacerbations are complex events that negatively impact the health of the patient. Their severity can be very variable, requiring hospitalization in cases of moderate or severe events [2]. In France, an exacerbation with hospitalization is responsible for an average additional cost of approximately 8300 euros [3].

In addition, with each new exacerbation, the chances of further exacerbation and the risk of mortality increase [4].

Early management of exacerbations is essential to reduce mortality, limit the patient’s loss of ventilatory capacity and reduce hospitalisations and costs of health [5].

It is nowadays possible to use passive and non-invasive equipment to follow patients under oxygen therapy at home by measuring and recording a breathing signal. Using machine learning on such signals for an early detection of abnormality in the respiratory process could be a major challenge to improve the COPD patient care.

### Related work

Within this context, researchers have been looking for new technologies and methods for monitoring COPD patients with the intention of early identifying acute exacerbation events. Some methods, based on self-reported symptoms or manually entered data [6–10] are limited, since they depend on subjective assessment and on patient compliance. Others have been focusing in remote monitoring devices, enabling automatic follow-up of physiological data and reducing the need for intervention for data acquisition by patients or the health team [11–16].

Some of the methods described employ an online learning process, that can be considered as a novelty detection approach. Sometimes called one-class classification, the novelty detection consists of describing a “normality” class, from which new points can be classified as belonging or not. They are often used in medical problems modelling, in which a lot of the data belongs to “normality” while the “abnormal” events not only are rare, but also variable, meaning that the characteristics of abnormality may not be known a priori [17, 18].

In the case of exacerbation prediction, one frequent hypothesis is that changes in the breathing pattern may occur before exacerbation. More specifically, some authors have found that a significant change in breathing rate may be related to an exacerbation event [11, 13, 14, 16].

Among the remote monitoring devices proposed, some have the particularity of being coupled with a non-pharmacological treatment. That is the case of some non-invasive ventilation (NIV) machines [11, 14, 16] that allow monitoring with minimal patient effort, since it only depends on patients treatment compliance. In the most recent of these studies, a model for prediction of exacerbations based only on the respiratory rate performed with 93.5% sensitivity and 64.8% specificity. The model performance was increased when combining breathing rate with other measures from NIV [16].

With the same principle, other methods were proposed based on data from devices that monitor patients under long-term oxygen therapy (LTOT) [13]. Compared to NIV, no mask is used, the breathing is only spontaneous and measures concern only the nasal pressure. In the latter study, an increase in breathing rate was able to predict exacerbations with 66% sensitivity and 93% specificity. LTOT is the most used non-pharmacological treatment among patients with COPD in France [19]. Therefore LTOT monitoring devices allow to cover another part of the population, while capturing measures from patient’s spontaneous breathing. Nonetheless, those devices are for now limited to the monitoring of breathing rate and treatment compliance.

The TeleOx^*Ⓡ*^ (Srett, Boulogne-Billancourt, France) is a medical device designed to evaluate adherence and treatment efficacy in LTOT patients. The device is placed on the oxygen circuit between the source and the nasal cannula of the patient, adding no new constraints for the patient. Initially developed to follow patients compliance to treatment, TeleOx^*Ⓡ*^ also enables monitoring flow rate of oxygen and the respiratory rate of the patient at regular intervals (45 seconds every 5 minutes) [20].

As described in [20], TeleOx^*Ⓡ*^ data is computed by associating a pressure sensor and a fluidic oscillator flow sensor. From those sensors, TeleOx^*Ⓡ*^ measures a signal that corresponds to patients’ nasal pressure, which can be used to compute a proxy of the patients oxygen flow and respiratory rates. These parameters are recorded in the device memory for further upload in a server. The recorded data show a higher level of noise than respiratory data at hospital but allow to follow patients at home, with no invasive device nor manipulation needed.

### Aim of the present article

Predicting exacerbations is widely used as the main objective in clinical studies. Yet, this is a difficult outcome to monitor, as there is no consensus in the definition of an exacerbation. Moreover, it requires a long term follow-up and shows high variability between patients.

Since exacerbations correspond to an imbalance between the respiratory muscle load-capacity-drive relationship [21], we look for another way of analysing how this balance reflects in breathing and detecting changes in a shorter-term follow-up, using the TeleOx^*Ⓡ*^ device.

In its stable state, a patient with COPD has a precarious load-capacity balance. Its basal load level is already high because of increased airway resistance and decreased dynamic chest wall elastance. To compensate this excess load, the respiratory muscles of these patients are highly demanded. In addition, COPD often comes with muscle weakness, which reduces the ability of the respiratory muscles to compensate for this load. COPD patients are therefore exposed to a significant risk of imbalance.

During an episode of acute respiratory infection, the increase, even moderate, of the respiratory load may be greater than the compensatory capacity of the respiratory muscles, already in high demand in the basic state. This decompensation generates an increase in symptoms such as dyspnea and coughs and a reduction in oxygen saturation.

In healthy subjects, the balance between respiratory load and compensatory capacity has more potential to adapt to different situations. At rest, a small proportion of the capacity of the respiratory muscles is sufficient to compensate for the low breathing loads. During an increase in the respiratory load (pneumonia, asthma attack, physical effort, etc.), the activity of the respiratory muscles can be increased without exceeding their maximum capacity.

Therefore, even for a healthy individual, a change in the load-capacity-drive balance involves changes in the way he or she breathes. Thus, the prediction of exercising may be used as a proof-of-concept problem before looking at decompensations in patients with COPD. This paper focuses on the use of machine learning techniques in order to (a) identify features that well describe respiratory pattern changes in healthy individuals using annotated data, and (b) verify if those same features enable to identify respiratory pattern changes in patients with COPD.

To do so, we compare the use of the breathing rate alone with the couple breathing rate-breathing amplitude which is more representative of the subject’s breathing load. We also compare them to more complex and standard feature extraction methods for time series data which are ARIMA models and Fourier decomposition. This comparison is made in terms of prediction capability using generalized linear mixed effect models.

In a second time, we provide a proof-of-concept procedure to show the ability of the selected features to detect abnormality. To do so, we apply a one-class classification method, that is, we train a model only on resting data and evaluate its ability to predict exercising.

## Results

### Data and feature extraction

In total, dataset from twenty healthy subjects contained 439 rest periods and 78 effort periods. In the COPD dataset, 1567 rest periods and 571 effort periods were recorded from eight patients.

**Fig. 1:**
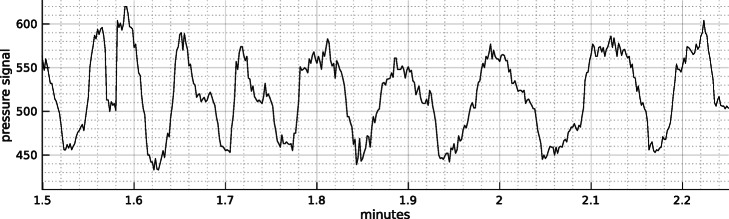
Example of pressure signal recorded with TeleOx^*Ⓡ*^. Window of 45 seconds of nasal pressure signal from a healthy subject recording

In Fig. 1, we present an example of what a 45 seconds period of recording by TeleOx^*Ⓡ*^ can look like for a healthy subject at rest. This signal, although not exactly corresponding to the respiration, is used as a proxy of it to extract the features.

**Fig. 2:**
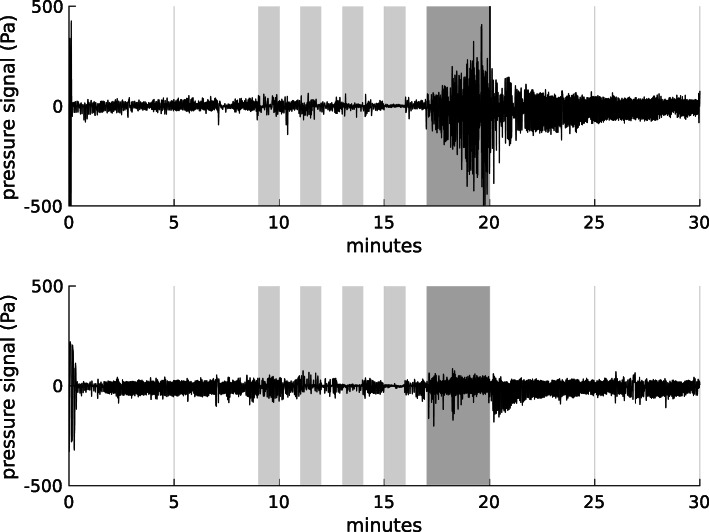
Full pressure signal TeleOx^*Ⓡ*^ recordings for two healthy subjects. Dark gray areas correspond to the 3 minutes of exercising while the light gray areas correspond to breathing while drinking, coughing, speaking and oral breathing. Subjects were at rest in other areas

Figure 2 presents two examples of healthy nasal pressure recordings. The dark gray area corresponds to the 3 minutes of exercising. One can see on those examples that the strategy adopted to increase the respiratory load is different from one person to another. The top individual modifies the amplitude of his breathing, while the second increases his breathing rate. This very different behavior justifies the use of models for which the classification rules are learned individual per individual, rather than in common.

**Fig. 3:**
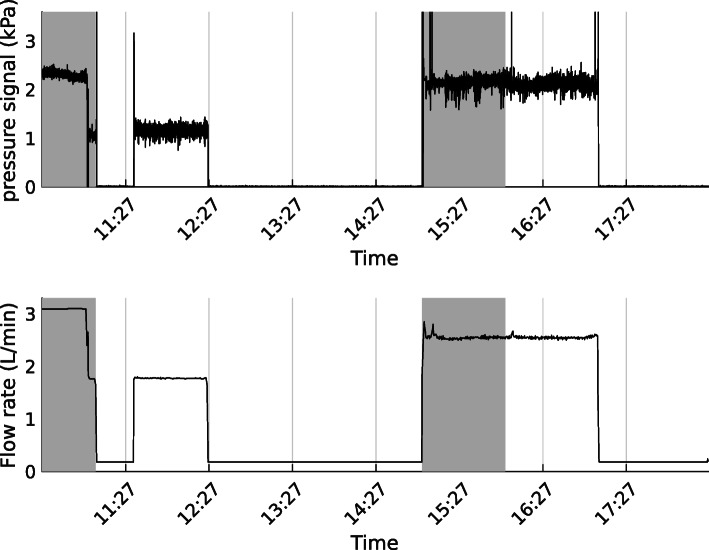
Pressure signal and oxygen flow for 8-hours recording from COPD patient. Gray areas correspond to estimated exercise times

One example of recording for a COPD patient is given in Fig. 3. Oxygen flow is not used in the analysis but it allows for understanding variations in the pressure signal. Only periods where continuous oxygen flow is detected are used in following analysis.

**Fig. 4:**
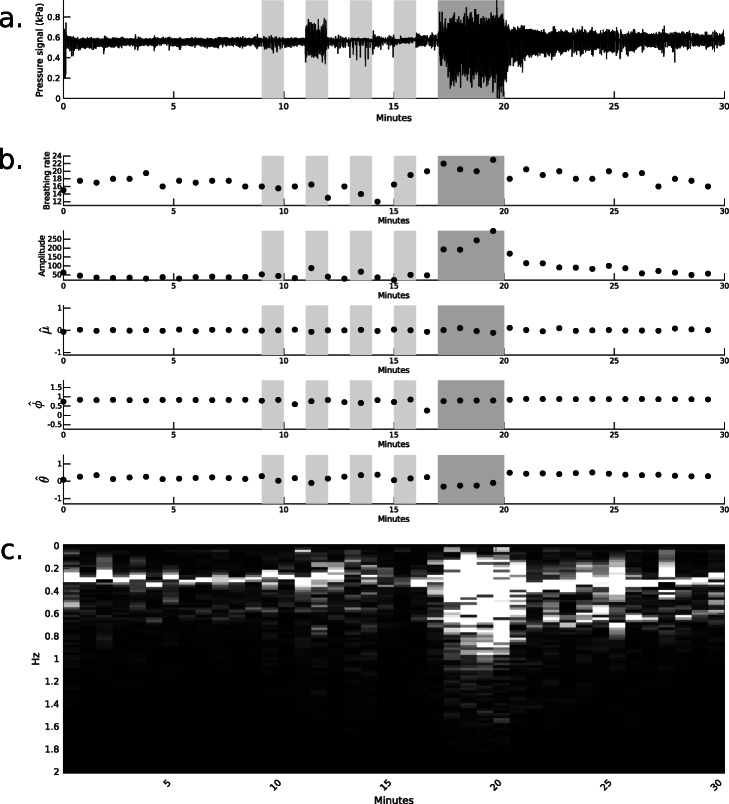
Extracted features example from a healthy subject recording. **a** Raw pressure signal, **b** breathing rate, signal amplitude and ARIMA coefficients and **c** Fourier transform. In **a** and **b**, dark gray areas correspond to the 3 minutes of exercising while the light gray areas correspond to breathing while drinking, coughing, speaking and mouth breathing

**Fig. 5:**
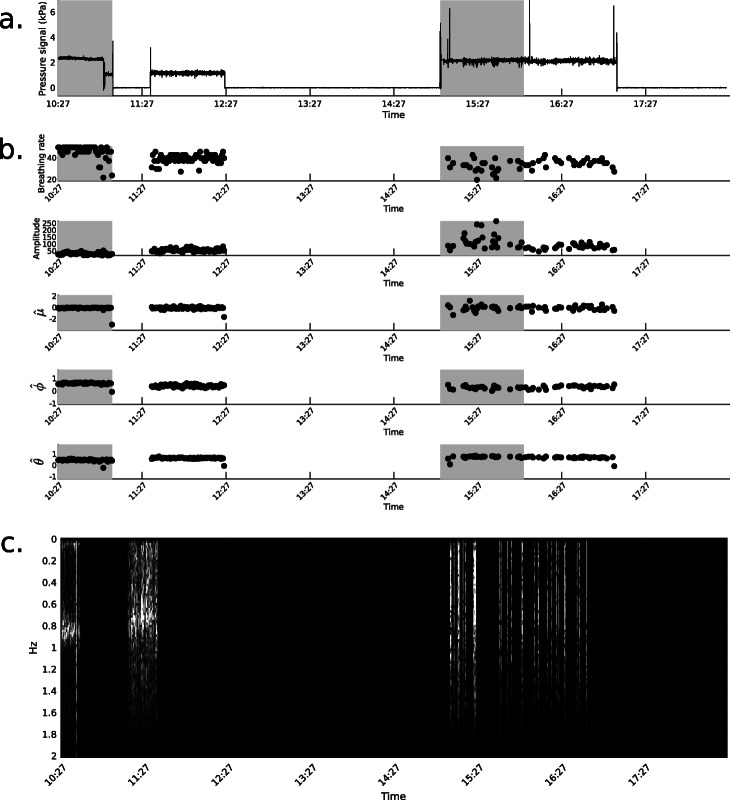
Extracted features example from a COPD patient recording. **a** Raw pressure signal, **b** breathing rate, signal amplitude and ARIMA coefficients and **c**. Fourier transform. In **a** and **b**, dark gray areas correspond to estimated exercise times

Figures 4 and 5 show examples from healthy and COPD recordings, respectively, and the corresponding features computed every 45 s. COPD recordings often include periods where features are not computed, which correspond to periods where oxygen is not used, patient is not detected or the quality of the signal is considered insufficient.

### Supervised classification

**Fig. 6:**
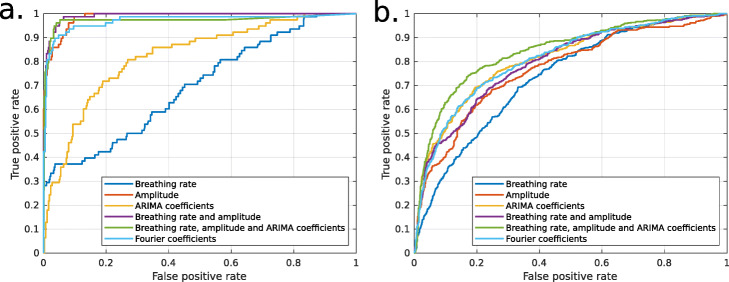
ROC curves for the detection of exercise periods in the supervised context using combinations of the proposed features. **a** Healthy subjects and **b** Patients with COPD

Classification methods were performed with healthy and COPD datasets separately. Generalized linear mixed models (GLMM) performances are presented in Tables 1 and 2 for healthy and COPD datasets respectively. Figure 6 shows the ROC curves for the different combinations of features in both cases.

**Table 1:** Performance of supervised classification models in exercise detection for the healthy individuals dataset using different predictor variables and performance indices

Predictive variables	Accuracy	Sensitivity	Specificity	AUC
Breathing rate	0.886	0.993	0.282	0.734 (0.673-0.794)
Signal amplitude	0.957	0.986	0.795	0.987 (0.978-0.995)
ARIMA coefficients	0.859	0.959	0.295	0.820 (0.769-0.872)
Breathing rate and signal amplitude	0.965	0.984	0.859	0.995 (0.991-1.000)
Breathing rate, signal amplitude and ARIMA coefficients	0.963	0.979	0.872	0.977 (0.945-1.000)
Fourier coefficients (frequencies ≤2 Hz)	0.954	0.973	0.846	0.975 (0.948-1.000)

**Table 2:** Performance of supervised classification models in exercise detection for the COPD patients dataset using different predictor variables and performance indices

Predictive variables	Accuracy	Sensitivity	Specificity	AUC
Breathing rate	0.748	0.950	0.194	0.741 (0.718-0.764)
Signal amplitude	0.787	0.951	0.338	0.773 (0.751-0.796)
ARIMA coefficients	0.806	0.945	0.424	0.814 (0.793-0.835)
Breathing rate and signal amplitude	0.801	0.939	0.422	0.798 (0.776-0.819)
Breathing rate, signal amplitude and ARIMA coefficients	0.825	0.932	0.531	0.848 (0.829-0.867)
Fourier coefficients (frequencies ≤2 Hz)	0.797	0.933	0.422	0.811 (0.791-0.832)

In the healthy dataset, the comparison between methods shows a clear hierarchy in their capacity to discriminate rest and exercise. Breathing rate and ARIMA coefficients alone are clearly weaker than their combinations with amplitude or Fourier coefficients. In the COPD dataset, the combination of breathing rate, amplitude and ARIMA coefficients is superior to any other case tested.

There is also a clear difference between healthy individuals and COPD patients but it is difficult to tell if it is due to COPD or to the lesser confidence of the data labeling.

### One-class classification

**Fig. 7:**
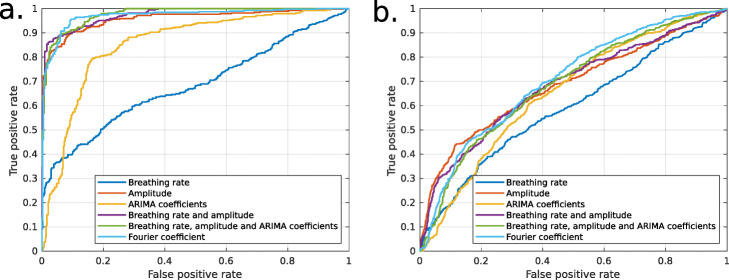
ROC curves for the detection of exercise periods in the one-class context using combinations of the proposed features **a** Healthy subjects and **b** Patients with COPD

ROC curves of the results of one-class classification models based on the Mahalanobis distance are shown in Fig. 7. The performances obtained by each method are presented in Tables 3 and 4 for healthy and COPD datasets, respectively.

**Table 3:** Performance of one-class classification models in exercise detection for the healthy individuals dataset using different predictor variables and performance indices

Predictive variables	Accuracy	Sensitivity	Specificity	AUC
Breathing rate	0.655	0.597	0.706	0.684 (0.647-0.721)
Signal amplitude	0.905	0.903	0.907	0.958 (0.942-0.971)
ARIMA coefficients	0.817	0.795	0.836	0.855 (0.828-0.880)
Breathing rate and signal amplitude	0.918	0.887	0.945	0.974 (0.964-0.981)
Breathing rate, signal amplitude and ARIMA coefficients	0.919	0.895	0.941	0.976 (0.967-0.983)
Fourier coefficients (frequencies ≤2 Hz)	0.929	0.951	0.909	0.971 (0.957-0.979)

**Table 4:** Performance of one-class classification models in exercise detection for the COPD patients dataset using different predictor variables and performance indices

Predictive variables	Accuracy	Sensitivity	Specificity	AUC
Breathing rate	0.594	0.537	0.615	0.592 (0.561-0.619)
Signal amplitude	0.678	0.577	0.715	0.685 (0.655-0.709)
ARIMA coefficients	0.634	0.610	0.642	0.654 (0.627-0.678)
Breathing rate and signal amplitude	0.650	0.629	0.658	0.683 (0.656-0.712)
Breathing rate, signal amplitude and ARIMA coefficients	0.644	0.629	0.649	0.686 (0.661-0.711)
Fourier coefficients (frequencies ≤2 Hz)	0.655	0.639	0.662	0.705 (0.681-0.731)

In most tested conditions, the performances are slightly weakened compared to the supervised context. This was expected, since this method learns only about normal events, while supervised methods have access to both normal and abnormal events to learn the classification rules. The hierarchy between the proposed methods remains however similar, although the superiority of the combination of breathing rate, amplitude and ARIMA coefficient for the COPD dataset disappears.

## Discussion

The breathing rate alone has the lowest performance for classifying rest and effort periods for healthy individuals. Any other feature alone or combined leads to better performance among the considered models. The models combining breathing rate with amplitude, breathing rate with amplitude and ARIMA coefficients and Fourier coefficients present comparable and better performances.

Performances with data from patients with COPD are lower. Indeed, the periods labels are not as precise as for the healthy individuals dataset. Not only the time schedule is approximate, but also these patients may take exercising easier or execute physical activities in the rest periods. For some of those patients, any movement can become really challenging and be a physical effort, as walking, standing up, showering, etc.

In both cases, this study demonstrates a significant gain in combining breathing rate with amplitude and potentially ARIMA coefficients, or using Fourier decompositions. This gain can be measured in either Accuracy, Sensitivity/Specificity or AUC, and can be medically interpreted by the fact that changing one’s breathing rate is not the only way to adapt to changes in the load-capacity balance.

The supervised approach however has a major pitfall, which is that it needs abnormal data to learn the classification rules. In practice, two options are then available: learn rules about the entire population, or monitor patients for a sufficiently long period so several abnormal events are observed for each patient. The first solution is not relevant from a physiological point of view because it disregards the variability between patients. The latter is not attainable, since exacerbations arrive at different frequencies and may differ from one another, even for the same patient.

A novelty detection method, or one-class classification, may thus be preferred in the case of medical data. Normal data acquisition for each individual then allows to detect any possible change in the features. For instance, the second healthy subject presented in the Fig. 2 increases mainly its breathing rate during the exercising periods. Nevertheless, as soon as the subject sits down, there is an increase in the signal amplitude, a change not described previously. If the model is trained in a supervised manner with rest and effort periods, those first periods after exercising may be considered as rest.

On healthy subjects, the performances obtained using the Mahalanobis distance based model are similar to those obtained with GLMM, although a bit weaker for some of the predictors.

For the patients with COPD, the loss in performance is greater. Prediction with breathing rate alone achieves the lowest performances. Besides, in the supervised context, the model created by combining breathing rate, amplitude and ARIMA coefficients presented a superior performance than with any other predictive variable. In the unsupervised learning case, this combination is similar to other tested conditions. A slightly higher performance is obtained when using Fourier coefficients, which combines signal’s frequency, magnitude and variability.

It is also notable that, although breathing rate alone classifies better than random guessing for both healthy and COPD subjects, its performance is quite poor in all cases as previously described. Combining the breathing rate with any other feature described here seems to be beneficial for the identification of periods of exercising. This observation is again true for all considered classification criteria, that is Accuracy, Sensitivity/Specificity or AUC.

## Conclusions

The conclusion of this study is that breathing rate alone is a quite poor feature in terms of prediction of breathing change, in the sense that the addition of any of the other proposed features clearly improves the classification power, in both supervised and novelty detection framework. From this point of view, the most promising features are the Fourier coefficients and the combinations of breathing rate with other predictive variables, notably the signal amplitude.

Besides, although this study do not consider breathing change patterns specifically related to exacerbation events, these new features may also enlighten other kinds of load-capacity balance changes and thus potentially enhance exacerbation prediction methods.

## Methods

### Monitoring device

TeleOx^*Ⓡ*^ (Srett, Boulogne-Billancourt, France) firmware was adapted so the signals recorded by the pressure sensor and the fluidic oscillator flow sensor are kept with the computed parameters. Signals are recorded at 10 Hz. This manipulation limits the data acquisition to eight hours of recording, after which all data must be erased before a new data acquisition.

### Data

Data was collected from a set of twenty healthy subjects and eight patients with COPD from two different protocols.

Healthy subjects study protocol consisted of simulating oxygen therapy by replacing the oxygen source by an air source. Each subject followed instructions for a total of 30 minutes, executing activities as: resting, drinking, coughing, speaking, mouth breathing, exercising and recovering, at given time intervals. Raw pressure and flow signals were recorded with TeleOx^*Ⓡ*^. All participants provided written informed consent for study participation.

Participants with COPD were recorded over a period of eight hours in a purely observational manner. The patients were enrolled while hospitalized at the Service de Pneumologie, Médecine Intensive et Réanimation, Groupe Hospitalier Pitié-Salpêtrière. The inclusion criteria required that patients had COPD, were under oxygen therapy and already monitored with a TeleOx^*Ⓡ*^. The regular TeleOx^*Ⓡ*^ was replaced by a TeleOx^*Ⓡ*^ with the new firmware in the morning (around 10 am). Eight hours later, the TeleOx^*Ⓡ*^ were recovered and the regular TeleOx^*Ⓡ*^ were plugged back. Patients followed the Unit predefined schedule, including a bike session in the morning and supervised gymnastics in the afternoon. This allowed an estimation of the periods of exercising during the day.

### Feature extraction

Signal treatment was based on the TeleOx^*Ⓡ*^ original algorithm. Therefore, features were extracted considering 45 seconds windows and features were only computed when subject presence was identified.

This means that, for each healthy subject, there were 40 windows. For COPD patients, the number of windows is variable, according to patients adherence to oxygen therapy.

Periods with less than 4 identified breaths or with breathing lengths too variable were considered as poor quality periods and ignored from analysis.

The following paragraphs detail the features which prediction power are compared, isolated or combined.

#### Breathing rate

Breathing rate is a commonly used feature for monitoring patients with COPD. As described previously, it has already been shown that there is a correlation between breathing rate increase and forecoming exacerbation.

Breathing rate is computed as the inverse of the median breathing length in the 45 seconds period. This feature calculation has been validated in a previous paper [20].

#### Amplitude

More than a change in breathing rate, a visual analysis of healthy records shows a significant change in signal amplitude during different moments of recordings. The amplitude is computed as the median amplitude at inspiration, which corresponds to the distance between pressure signal minima and the estimated baseline.

#### Fourier coefficients

Another way of analysing frequency, amplitude and variability of the signals is by using Fourier transforms. The Fourier decomposition makes it possible to analyze periodic functions by describing their frequency spectrum, which means that it highlights the frequencies present in the signal and their respective magnitude.

The Fourier coefficients of the function *f* are given by :

1 $$\begin{array}{@{}rcl@{}} c_{n} = 1/T \int_{0}^{T}f(t) e^{-in\omega t}dt \end{array} $$

Where *T* is the period, *ω*=2*π*/*T* is the pulsation of *T* and the magnitudes are given by the absolute values of *c*_*n*_.

We chose to only keep frequencies bellow 2 Hz to limit the number of features. Besides, a breathing rate of 2 Hz already has no physiological sense, so we can consider that above this frequency we would only be analyzing noise.

#### ARIMA coefficients

ARIMA is a modelisation approach for time series data relying on the assumption that the signal is autoregressive, that is the value at each time point can be written as resulting from a linear model according to the preceding points and their errors [22].

We use the ARIMA model (1,1,1), which follows the following prediction equation:

2 $$\begin{array}{@{}rcl@{}} y_{t} = \mu+y_{t-1}+\Phi \cdot (y_{t-1}-y_{t-2})-\theta \cdot \epsilon_{t-1}+\epsilon_{t} \end{array} $$

So, for every 45 seconds of recording, we estimate the parameters $(\hat {\mu },\hat {\Phi },\hat {\theta }) $ which describe the temporal dynamics of the signal, where *μ* is the signal constant, *Φ* is the auto-regression coefficient, *θ* is the moving average coefficient of the model and *ε* is the random error related to the observation.

### Classification data

For healthy subjects, a total of 40 data points are extracted, corresponding to non-overlapping sequences of 45 seconds of raw data. Each of those sequences is transformed in a vectorial data by extracting the features described in the previous subsection.

Among those 40 points, the first 23 are considered as rest/reference. Drinking, coughing, speaking and mouth breathing are included in the reference class, since those events are expected to happen as the “normality” for patients with COPD. The following 4 points correspond to exercising. The last 13 points are a transition between effort and rest and thus are not used for classification methods.

For the recordings from patients with COPD, the labels are not that clear. The approximate periods of exercise (bike session and supervised gymnastics) belong to the exercise class. All other data points are considered as reference. Like the healthy data, the reference certainly includes drinking, coughing, speaking and mouth breathing, as well as other events as eating, moving and walking, which are unknown.

### Supervised classification

To take into account the inter-individual variability, generalized linear mixed effect models (GLMM) were used in the supervised context. Different combinations of features were tested so the ability of classifying rest and effort breathing can be compared. Healthy and COPD datasets are trained and tested separately.

Models are built using *glmer* function of the *lme4* package [23] in R, with random intercepts and random slopes. The link function is logit.

The Fourier coefficients cannot be used directly because of the high number of coefficients. To avoid the curse of dimensionality, we perform for each fold a PCA on the training data and keep the first 5 principal components.

### One-class classification

The supervised classification power is not the only criterion to select a combination of features. Indeed, in the objective application, the algorithm will have to train based only on a normal class and be able to detect abnormalities.

We choose to compare the abilities for such a task of the features listed in “Feature extraction” section by using a method based on Mahalanobis distance [18, 24]. Given training data of mean *μ* and variance-covariance matrix *Σ*, the Mahalanobis distance of a new measure *x* to the training data is defined by the following equation.

3 $$\begin{array}{@{}rcl@{}} d (x) = \left[(x-\mu)'\Sigma^{-1}(x-\mu)\right]^{1/2} \end{array} $$

**Fig. 8:**
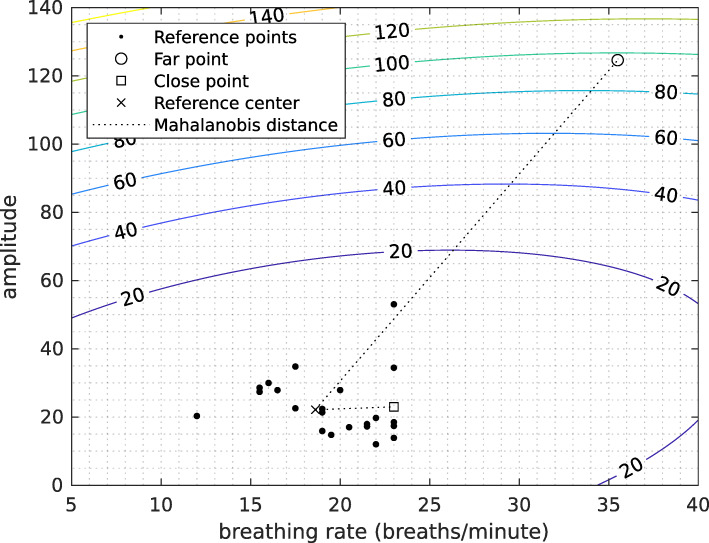
Example of Mahalanobis distances from reference points. In the one-class context, distances between each new measure *x* and the all reference points are given by the Mahalanobis distance, considering the reference’s mean and variance-covariance matrix

The underlying idea is to consider that reference data are spread according to a multidimensional normal law (which shape is given by the variance-covariance matrix) and that the distance grows as the distribution of that law decreases. Figure 8 presents an example of the Mahalanobis distance from a reference distribution in the plane breathing rate-amplitude.

For each subject, we divide the data points into training set and validation set. The training set contains only rest points and is used as the reference. The validation set contains both rest and effort points, for which we compute the Mahalanobis distance from the training set. A 5-fold cross-validation method is used to both data from healthy subjects and from patients with COPD. For each subject, healthy or COPD patients, a 5-fold cross validation is used on the resting data: 4 out of the 5 folds are used to learn the mean and variance-covariance matrix.

For the healthy dataset, the distance is computed for the last fold and the exercising data, ending up with one prediction for each resting point and five predictions for each exercising point. For the COPD dataset, larger exercising data is available, the distance is thus computed for the last fold of resting data and one fold of the exercising data. We thus end up with one prediction for each data point (rest and exercise).

This method is repeated varying the features used: 1. breathing rate; 2. signal amplitude; 3. breathing rate and amplitude; 4. ARIMA coefficients; 5. breathing rate, amplitude and ARIMA coefficients and 6. Fourier coefficients.

The dimensionality of Fourier coefficients also needed to be reduced for the one-class classification method. For each subject, a PCA using training resting data was used to define the first 5 principal components. Mahalanobis distance and the method described above is then completed using the projected data.

Sensitivity and specificity is given for the cut-off threshold that minimizes the distance from the upper-left corner of the respective ROC curve, that is $\sqrt {FPR^{2}+(1+TPR)^{2}}$, where FPR is the false positive rate and TPR is the true positive rate.

## Data Availability

The datasets used and/or analysed during the current study are available from the corresponding author on reasonable request.

## Abbreviations

ARIMA: Autoregressive integrated moving average
COPD: Chronic obstructive pulmonary disease
LTOT: Long-term oxygen therapy
NIV: Non-invasive ventilation
